# Comparative study of hospitalized children with acute respiratory distress syndrome caused by SARS-CoV-2 and influenza virus

**DOI:** 10.1186/s12879-021-06068-w

**Published:** 2021-05-04

**Authors:** Xinghua Liu, Wei Li, Bo Zhang, Yan Guo, Zhao Hu, Cao Peng, Xiao Lei, Qunying Luo, Qiong Zhang, Wei Deng, Juanjuan Wang, Jianqiao Tang, Yunqiao Li, Jianying Chen

**Affiliations:** 1 Department of Gastrointestinal Surgery, Union Hospital, Tongji Medical College, Huazhong University of Science and Technology, Wuhan, 430022 China; 2 Department of Thyroid and Breast Surgery, Union Hospital Affiliated to Tongji Medical College, Huazhong University of Science and Technology, Wuhan, 430022 China; 3 Wuhan Center for Disease Control and Prevention, Wuhan, 430024 China; 4 Department of Rheumatology and Immunology, Wuhan Children’s Hospital, Tongji Medical College, Huazhong University of Science and Technology, Wuhan, 430016 China; 5 Department of Emergency Medicine, Union Hospital, Tongji Medical College, Huazhong University of Science and Technology, Wuhan, 430022 China; 6 Department of Geriatrics, Union Hospital, Tongji Medical College, Huazhong University of Science and Technology, Wuhan, 430022 China; 7 Department of Rheumatology and Immunology, Anhui Provincial Children’s Hospital, Hefei, 230032 China; 8 Department of Pediatrics, Wuhan Children’s Hospital, Tongji Medical College, Huazhong University of Science and Technology, Wuhan, 430016 China

**Keywords:** Children, Clinical features, COVID-19, Influenza, SARS-CoV-2

## Abstract

****Background**:**

Since the outbreak of coronavirus disease 2019 in December 2019, more than 8 million cases have occurred worldwide as of June 16, 2020. However, it is important to distinguish COVID-19 from other respiratory infectious diseases, such as influenza. Here, we comparatively described the clinical characteristics of children with COVID-19 and paediatric patients with influenza.

**Methods:**

In this retrospective, single-centre study, we reviewed the electronic medical records of 585 paediatric patients with COVID-19 or influenza in Wuhan Children’s Hospital, China. Clinical and epidemiological characteristics, laboratory findings, and clinical outcomes were comparatively analysed.

**Results:**

The median ages were 6.96 years (IQR, 2–10.81) for children with confirmed COVID-19, 2.67 years (IQR, 1.03–15.25) for those with influenza A and 3.67 years (IQR, 1.62–5.54) for those with influenza B. Fever was a symptom in 84 (34.7%) COVID-19 cases, 132 (70.21%) influenza A cases and 111 (74.50%) influenza B cases. The median length of stay (LOS) was 11 (8–15) days for paediatric COVID-19 patients, 4 (3–6) days for influenza A patients and 5 (3–6) days for influenza B patients. Twenty-six (13.98%) influenza A patients and 18 (12.59%) influenza B patients presented with decreased white blood cell counts, while 13 (5.33%) COVID-19 patients presented with decreased white blood cell counts. Eight (3.28%) COVID-19 patients, 23 (12.71%) influenza A patients and 21 (14.79%) influenza B patients experienced lymphocytopenia. Acute cardiac injury occurred in 18 (7.29%) COVID-19 patients, while 37 (19.68%) influenza A and 27 (18.12%) influenza B patients had acute cardiac injury.

**Conclusion:**

In this study, the illnesses of children with COVID-19 were demonstrated to be less severe than those of paediatric patients with influenza, and COVID-19 patients had milder illness and fewer complications.

## Background

Coronavirus disease 2019 (COVID-19), which first emerged in Wuhan in December 2019 and is caused by severe acute respiratory syndrome coronavirus 2 (SARS-CoV-2), has rapidly spread throughout China and has spread worldwide in the past three months. As of March 28, 2020, SARS-CoV-2 has spread to most regions of China, with more than 82 thousand confirmed infections, and has spread to at least 190 countries with more than 2.7 million infected individuals. The number of COVID-19 cases in children was described as very small [1–3]. The data from the Chinese Centre for Disease Control and Prevention showed that out of 72,314 confirmed COVID-19 cases, there were 965 (1.3%) paediatric patients [2], and in another study from the United States, 2572 (1.7%) paediatric patients were reported among 149,082 confirmed cases [4]. However, with the continuous spread of SARS-CoV-2 worldwide, an increasing number of children will be infected in the future.

Recent studies have shown the primary features of paediatric patients with SARS-CoV-2 infection, and most paediatric cases appeared to be mild with symptoms of respiratory tract infection [5–7]. It has been difficult for clinicians to distinguish COVID-19 from other various respiratory diseases based on the symptoms experienced by children. Respiratory virus infections, such as influenza infection, respiratory syncytial virus infection and rhinovirus infection, are very common diseases among children, especially in the winter season [8]. Influenza was more common among children than adults, and 60% of patients were 18 years old or younger in the 2009 influenza pandemic [9]. In China, children seldom receive flu vaccination. Recently, global influenza-associated mortality climbed to the highest level compared with that in any other flu season, and more than 240,000 influenza cases, including influenza A virus (53.5%) and influenza B virus (46.5%) infection, were laboratory confirmed in the United States in the past season [10]. Hospitalization and mortality rates of influenza in children were higher than those previously reported [10]. Because of the mild illness experienced by paediatric COVID-19 patients, ailing children were very easily considered to have influenza, especially in the early period of the COVID-19 epidemic, and could not be admitted to the hospital in time. Although the illnesses of most children with COVID-19 were not severe, patients with milder illness and those without symptoms might play an important role in SARS-CoV-2 transmission. It is critical to distinguish COVID-19 from influenza in children. Here, we described the comprehensive clinical characteristics of children with COVID-19 and influenza during the COVID-19 epidemic in Wuhan.

## Methods

### Study design and participants

A total of 585 paediatric patients, including 248 with confirmed cases of COVID-19, 188 with confirmed cases of influenza A and 149 with confirmed cases of influenza B, from Wuhan Children’s Hospital were retrospectively studied. COVID-19 patients were hospitalized from January 26 to March 23, 2020, and influenza patients were admitted to the hospital from August 8, 2019, to January 26, 2020. All patients with confirmed COVID-19 recruited in this study were diagnosed by SARS-CoV-2 positivity in RT-PCR according to the World Health Organization interim guidance, and patients with confirmed influenza were diagnosed by the presence of IgM antibody against influenza virus A or B. The clinical information, including treatments, discharge dates and length of stay, was monitored up to March 28, 2020.

### Data collection

Data on epidemiological information, demographic characteristics, clinical manifestations, laboratory results, treatment measures and discharge date were extracted from electronic medical records. Clinical features consisted of the dates of illness onset and admission, symptoms, coexisting conditions and complications. Laboratory tests included a complete blood count, coagulation parameters, C-reactive protein, serum amyloid protein, procalcitonin, liver and renal function parameters, lactate dehydrogenase, lactate dehydrogenase isoenzyme 1, creatine kinase and creatine kinase isoenzyme 1. Treatment measures included oxygen therapy, antiviral agents, antibacterial agents, corticosteroids, budesonide suspension and immunomodulatory agents. All the clinical data were reviewed by team paediatricians. COVID-19 severity was classified according to experts’ consensus statement for COVID-19 in children [11]. Patients with asymptomatic cases were defined as children positive for SARS-CoV-2 without manifestations of clinical symptoms or abnormal chest imaging findings. Patients with mild cases were defined as patients who had only fever, cough, pharyngeal pain, nasal congestion, fatigue, headache, myalgia or discomfort but no abnormal radiological findings. Patients with moderate cases were defined as children who had pneumonia on chest CT with or without fever and respiratory symptoms such as cough but did not meet the criteria for severe pneumonia. Patients with severe cases were defined as those who met any of the following criteria: (1) increased respiratory rate: ≥ 70 times/min for those < 1 year of age or ≥ 50 times/min for those ≥ 1 year of age (after ruling out the effects of fever and crying); (2) oxygen saturation < 92%; (3) hypoxia: assisted breathing (moans, nasal flaring, and three-concave sign), cyanosis, or intermittent apnoea; (4) a disturbance of consciousness: somnolence, coma, or convulsion; and (5) food refusal or feeding difficulty, with signs of dehydration. Patients with critical cases met one of the following criteria and required intensive care unit (ICU) care: (1) respiratory failure requiring mechanical ventilation, (2) shock, or (3) other organ failure.

### SARS-CoV-2 nucleic acid testing

SARS-CoV-2 nucleic acid testing was performed by the laboratory department of Wuhan Children’s Hospital, and the procedure was the same as that described by Lu et al. [7]. In brief, nasopharyngeal or throat swabs were tested for the presence of SARS-CoV-2 nucleic acid using real-time reverse transcription polymerase chain reaction (RT-PCR). Viral nucleic acids were extracted with the High Pure Viral Nucleic Acid Kit (Zhongzhi, Wuhan, China). The primers for RT-PCR assays were as follows:

forward primer 5′-TCAGAATGCCAATCTCCCCAAC-3′;

reverse primer 5′-AAAGGTCCACCCGATACATTGA-3′; and.

probe 5′ CY5-CTAGTTACACTAGCCATCCTTACTGC-3′ BHQ1.

Amplifications were performed by incubation at 50 °C for 15 min and 95 °C for 3 min, followed by 45 cycles at 95 °C for 15 s and 60 °C for 30 s.

### Statistical analysis

Continuous variables were described as median and interquartile range (IQR) values. The comparison of medians was analysed with the Kruskal-Wallis test. Categorical variables were calculated as the percentages of patients in each category and compared using χ2 or Fisher’s exact tests as appropriate. All statistical analyses were performed in SPSS software version 20.0. Two-sided α values of less than 0.05 indicated statistical significance.

## Results

### Clinical characteristics of paediatric patients with COVID-19 and influenza

**Fig. 1:**
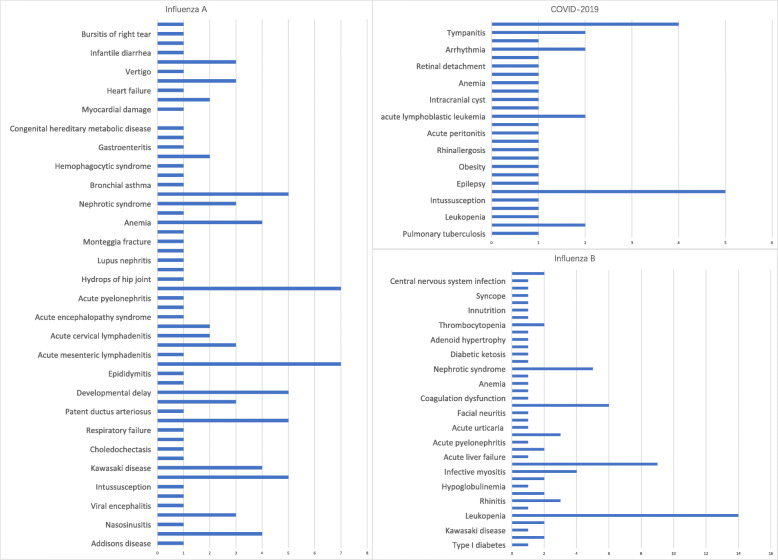
All coexisting conditions in the 585 children

This study recruited 248 paediatric patients with confirmed cases of COVID-19, 188 with confirmed cases of influenza A and 149 with confirmed cases of influenza B. The median ages were 6.96 years (IQR, 2–10.81) for COVID-19 patients, 2.67 years (IQR, 1.03–15.25) for influenza A patients and 3.67 years (IQR, 1.62–5.54) for influenza B patients. The sex ratio of males to females was close to 1.5 in the three groups. Familial clustering was defined based on family infection history, which was recorded in the medical record, and familial clustering of infection occurred in 192 (77.4%) COVID-19 patients (Table 1). Among confirmed COVID-19 cases, 39 (15.7%) patients had coexisting conditions, while the number of children with comorbidities was 67 (35.64%) in the influenza A group and 41 (27.52%) in the influenza B group. The most common comorbidity was leukopenia; other comorbidities included hyperglobulinemia, acute lymphoblastic leukaemia, arrhythmia, tympanitis, central atrial septal defect, rhinitis, convulsion, Kawasaki disease, epilepsy, jaundice, atrial septal defect, epididymitis, acute tonsillitis, acute laryngitis, acute cervical lymphadenitis, acute gastroenteritis, anaemia, eczema, diabetic ketosis, myocarditis, thrombocytopenia, viral encephalitis, infective myositis, cerebral palsy, and thrombocytopenia (Fig. 1).

**Table 1:** Clinical Characteristics of pediatric patients infected with Covid-19, Influenza A and Influenza B

		Covid-19 (***N*** = 248)	Influenza A (***N*** = 188)	Influenza B (***N*** = 149)	***P*** Value
**Age**		6.96 (2–10.81)	2.67 (1.03–5.25)	3.67 (1.62–5.54)	< 0.0001
**Sex**	Male	152 (61.29%)	119 (63.30)	88 (59.06%)	0.730
	Female	96 (38.71%)	69 (36.70%)	61 (40.94%)	
**Signs and Symptoms**					
Temperature					< 0.0001
< 37.3 °C		162 (65.32%)	56 (29.79%)	38 (25.50%)	
37.3 °C–38.5 °C		56 (22.58%)	50 (26.60%)	46 (30.87%)	
38.5 °C–39.0 °C		10 (4.03%)	14 (7.45%)	14 (9.40%)	
> 39.0 °C		20 (8.06%)	68 (36.17%)	51 (34.23%)	
Cough		79 (31.85%)	146 (77.66%)	116 (77.85%)	< 0.0001
Diarrhea		7 (2.82%)	24 (12.77%)	5 (3.36%)	0.0001
Chills		2 (0.81%)	40 (21.28%)	24 (16.11%)	0.0024
Fatigue		3 (1.21%)	3 (1.60%)	1 (0.67%)	0.740
Vomit		7 (2.82%)	39 (20.74%)	25 (16.78%)	< 0.0001
Nasal congestion and Rhinorrhea		8 (3.23%)	57 (30.32%)	35 (23.49%)	< 0.0001
Muscle soreness		2 (0.81%)	1 (0.53%)	0	0.7359
Headache		2 (0.81%)	6 (3.19%)	4 (2.68%)	0.3128
Sneeze		2 (0.81%)	23 (12.23%)	10 (6.71%)	< 0.0001
Sore throat		3 (1.21%)	2 (1.06%)	2 (1.34%)	0.7502
Tachypnea		2 (0.81%)	6 (3.19%)	5 (3.36%)	0.004
abdominal pain		4 (1.61%)	10 (5.32%)	9 (6.04%)	0.1052
Rash		1 (0.40%)	5 (2.66%)	3 (2.01%)	0.0928
Poor spirits		4 (1.61%)	6 (3.19%)	10 (6.71%)	< 0.0001
Dyspnea		1 (0.40%)	5 (2.66%)	1 (0.67%)	0.0181
Asthma		1 (0.40%)	28 (14.89%)	16 (7.69%)	< 0.0001
Phlegm		1 (0.40%)	24 (12.77%)	24 (16.11%)	< 0.0001
**Type of Severity of Illness**					
Asymptomatic		72 (29.13%)			< 0.0001
Mild and Moderate cases		172 (69.36%)	171 (90.96%)	141 (94.63%)	
Severe cases		4 (1.61%)	17 (9.04%)	8 (5.37%)	
Days from illness onset to hospitalization (days)		5 (3–8)	4 (2–8)	4 (2.5–7)	0.0781
Days from hospitalization to Discharge (days)		11 (8–15)	4 (3–6)	5 (3–6)	< 0.0001
**Family member infection**					
no		56 (22.58%)			
yes		192 (77.42%)

Fever and cough were the most common symptoms in the three groups, and 86 (34.7%) COVID-19 patients presented with fever, compared to 132 (70.21%) influenza A patients and 111 (74.50%) influenza B patients, and the difference was statistically significant. For paediatric COVID-19 patients, nasal congestion, rhinorrhoea, diarrhoea and vomiting were other common symptoms, and abdominal pain and tachypnoea were less common symptoms. In the influenza groups, phlegm, asthma, sneezing, chills and vomiting were more frequently found as residual symptoms (Table 1). There were 172 (69.36%) mild or moderate COVID-19 cases, and 4 (1.61%) severe or critical cases; these numbers were 171 (90.96%) and 17 (9.04%), respectively, for influenza A and 141 (94.63%) and 8 (5.37%), respectively, for influenza B. In addition, the median duration from the onset of illness to admission was 5 (3–8) days for COVID-19 patients. The median length of stay (LOS) was 11 (8–15) days for paediatric patients with confirmed COVID-19 cases, 4 (3–6) days for those with confirmed influenza A cases and 5 (3–6) days for those with confirmed influenza B cases (Table 1).

### Laboratory findings of paediatric patients infected with SARS-CoV-2 and influenza virus

Twenty-six (13.98%) influenza A patients and 18 (12.59%) of influenza B patients had decreased white blood cell counts, while 13 (5.33%) COVID-19 patients had decreased white blood cell counts. Eight (3.28%) COVID-19 patients had lymphocytopenia, whereas 23 (12.71%) and 21 (14.79%) influenza A and influenza B patients, respectively, had lymphocytopenia. The percentage of COVID-19 patients with abnormally elevated myocardial enzymes was less than that of influenza paediatric patients (41.95% vs 65.06 and 62.60%, *p* < 0.0001). For inflammatory markers, the most common abnormities in the three groups were found in the erythrocyte sedimentation rate, hypersensitive C-reactive protein (hsCRP) and serum amyloid A. Serum amyloid A was increased in 13 (16.46%) COVID-19 patients, 64 (85.33%) influenza A patients and 46 (80.70%) influenza B patients (*p* < 0.0001), and hsCRP was abnormally elevated in 40 (18.60%) COVID-19 patients, 93 (56.71%) influenza A patients and 69 (40.46%) influenza B patients (*p* < 0.0001) (Table 2).

**Table 2:** Laboratory findings of pediatric patients infected with Covid-19, Influenza A and Influenza B

		Covid-19 (N = 248)	Influenza A (N = 188)	Influenza (N = 149)	P Value
**Blood routine**					
White blood cell count	Increase	23 (9.43%)	35 (18.82%)	27 (18.88%)	0.0001
	Decrease	13 (5.33%)	26 (13.98%)	18 (12.59%)	
Neutrophil count	Increase	33 (13.52%)	67 (37.02%)	39 (27.46%)	< 0.0001
	Decrease	13 (5.33%)	13 (7.18%)	10 (7.04%)	
Lymphocyte count	Increase	24 (9.84%)	21 (11.60%)	11 (7.75%)	0.0006
	Decrease	8 (3.28%)	23 (12.71%)	21 (14.79%)	
Hemoglobin (g/L; 110.0–140.0)		128 (120–137.75)	121.5 (112–128)	122 (113–129)	< 0.0001
Platelet (×10^9^/L; 100–320)		261.5 (208.5–314.75)	273 (209.5–363.25)	256 (202–337)	0.1707
Neutrophil/ Lymphocyte		1.06 (0.61–1.69)	1.22 (0.64–2.97)	1.31 (0.68–2.46)	0.0059
**Blood biochemistry**					
Total bilirubin	Increase	15 (6.22%)	11 (6.32%)	5 (3.76%)	0.5525
Albumin	Decrease	12 (4.98%)	25 (14.37%)	28 (13.53%)	0.0022
Alanine aminotransferase	Increase	27 (11.20%)	15 (8.62%)	7 (5.26%)	0.1536
γ-alkalinephosphatase	Increase	12(%4.98)	12 (6.90%)	7 (5.26%)	0.6683
Aspartate aminotransferase	Increase	32 (13.28%)	43 (24.71%)	40 (29.85%)	0.0003
Alkaline phosphatase	Increase	87 (36.10%)	31 (17.82%)	20 (15.04%)	< 0.0001
Blood urea nitrogen	Increase	3 (1.24%)	2 (1.18%)	1 (0.76%)	0.9091
Creatinine	Increase	12 (4.98%)	0	3 (2.29%)	0.0094
Lactate dehydrogenase L	Increase	54 (22.88%)	74 (44.58%)	58 (44.27%)	< 0.0001
Lactate dehydrogenase isozyme −1	Increase	64 (27.12%)	74 (44.58%)	59 (45.04%)	< 0.0001
Creatine kinase	Increase	15 (6.07%)	15 (8.02%)	14 (9.46%)	0.4487
Creatine kinase isoenzyme –MB	Increase	99 (41.95%)	108 (65.06%)	82 (62.60%)	< 0.0001
**Infection biomarkers**					
Hypersensitive troponin T	Increase	7 (5.93%)	6 (22.22%)	2 (15.38%)	0.0252
Ferroprotein	Increase	4 (2.82%)	6 (8.11%)	7 (12.50%)	0.0298
Serum amyloid A	Increase	13 (16.46%)	64 (85.33%)	46 (80.70%)	< 0.0001
Erythrocyte sedimentation rate	Increase	13 (22.81%)	46 (56.79%)	28 (51.85%)	< 0.0001
Hypersensitive C-reactive protein	Increase	40 (18.60%)	93 (56.71%)	69 (40.64%)	< 0.0001
Procalcitonin	Increase	5 (2.05%)	4 (2.23%)	1 (0.70%)	0.5328
D-Dimer	Increase	25 (13.81%)	22 (45.83%)	19 (52.77%)	< 0.0001
**Others pathogen infections**					
Mycoplasma		45 (40.91%)	46 (55.42%)	21 (42.00%)	0.1089
Cytomegalovirus		4 (3.03%)	6 (3.57%)	9 (7.03%)	0.2296
Eb virus		3 (1.99%)	7 (4.12%)	3 (2.24%)	04561
Influenza A		1 (0.91%)			
Influenza B		4 (3.63%)			
Legionella pneumoniae		0	1 (1.20%)	0	0.3799

No statistically significant difference was observed among the three groups in terms of comorbidities. SARS-CoV-2 co-infections with *Mycoplasma* (40.91%), cytomegalovirus (3.03%), and EB virus (1.99%) were observed, and the corresponding pathogens were detected in 55.42, 3.57 and 4.12% of influenza A patients and in 42.00, 7.03 and 2.24% of influenza B patients, respectively. There was one case of SARS-CoV-2 and influenza A virus co-infection and 4 cases of SARS-CoV-2 and influenza B virus co-infection (Table 2).

### Treatment and clinical outcome

Common treatments, including antiviral therapy, antibiotic therapy, the use of corticosteroids and immunoglobulin, aerosol inhalation of interferon-α and budesonide suspension, were sympathetically applied in the studied patients. Among COVID-19 patients, 50 (20.16%) were treated with antiviral drugs, 109 (43.95%) with antibiotic therapy, 235 (94.76%) with interferon-ɑ, and 3 (1.21%) with corticosteroids; 4 COVID-19 patients (1.61%) received intensive care. The percentages of children receiving corticosteroids and intensive care in the influenza group were obviously higher than those in the COVID-19 group, The median length of stay in ICU for children was: 21 days for COVID-19, 8.38 days for Flu A and 13 days for Flu B. Regarding oxygen support, in the COVID-19 group, 3 (1.21%) children required nasal cannulation and 4 (1.61%) children needed invasive mechanical ventilation, while 28 (14.89%) influenza A paediatric patients and 15 (18.12%) influenza B patients received oxygen support with nasal cannulation. The dysfunction of important organs was uncommon in COVID-19 patients. Acute cardiac injury occurred in 18 (7.29%) COVID-19 patents, while 37 (19.68%) influenza A and 27 (18.12%) influenza B patients experienced acute cardiac injury. In total, only one patient in this study died, who was reported to have a severe co-existing disease, intussusception [7], and most of the patients had favourable clinical outcomes (Table 3).

**Table 3:** Complications, Treatment and Outcome in pediatric patients infected with Covid-19, Influenza A and Influenza B

	Covid-19 (N = 248)	A influenza (N = 188)	B influenza (N = 149)	P Value
**Treatment**				
Antiviral therapy	50 (20.16%)	86 (45.74%)	65 (43.62%)	< 0.0001
Antibiotic therapy	109 (43.95%)	166 (88.30%)	143 (95.97%)	< 0.0001
Use of corticosteroid	3 (1.21%)	23 (12.23%)	13 (8.72%)	0.0001
Interferon α	235 (94.76%)	22 (11.70%)	13 (8.72)	< 0.0001
Immunoglobulin	1 (0.40%)	4((2.13%)	5 (3.36%)	0.0774
ICU	4 (1.61%)	14 (7.45%)	4 (2.68%)	0.0043
**Oxygen support**				
Nasal cannula	3 (1.21%)	28 (14.89%)	15 (10.07%)	< 0.0001
Invasive mechanical ventilation	4 (1.61%)	6 (3.19%)	2 (1.34%)	0.4164
**Complications**				
Cardiac injury	18 (7.26%)	37 (19.68%)	27 (18.12%)	0.0002
Liver dysfunction	13 (5.24%)	7 (3.72%)	3 (2.01%)	0.2725
Acute gastroenteritis	5 (2.02%)	5 (2.66%)	1 (0.67%)	0.4017
Neutropenia	0	8 (4.26%)	9 (6.04%)	0.0010
Lung injury	0	6 (3.19%)	0	0.0017
Renal injury	2 (0.81%)	1 (0.53%)	0	0.5520
MODS	2 (0.81%)	2 (1.06%)	0	0.4769
**Clinical outcome**				
Discharge or in hospital	247	188	149	
Death	1	0	0	

## Discussion

As of June 16, 2020, more than 8 million people worldwide have been infected with SARS-CoV-2, including more than 80 thousand people in China. However, approximately one thousand confirmed paediatric SARS-CoV-2 infections have been reported in China [2], and knowledge of the clinical features of paediatric cases is limited. Here, we comprehensively and comparatively characterized the clinical features of children with COVID-19 and those with influenza A or influenza B in Wuhan during the COVID-19 epidemic.

Our data demonstrated that the epidemiological profiles of paediatric COVID-19 patients were different from those of paediatric influenza patients. The age of children with COVID-19 was older than that of children with influenza. Our analysis showed that 46 (55.46%) influenza A patients and 21 (42.00%) influenza B patients were co-infected with *Mycoplasma*, 6 (3.54%) influenza A patients and 9 (7.03%) influenza B patients were co-infected with cytomegalovirus, and 7 (4.12%) influenza A patients and 3 (2.24%) influenza B patients were co-infected with Epstein-Barr virus. The distribution of these common pathogens in influenza patients was identical to that in COVID-19 patients. Few COVID-19 patients were co-infected with influenza. This might indicate that other pathogen infections were independent of SARS-CoV-2 infection. Similar to influenza [10, 12], SARS-CoV-2 exhibits prevalent human-to-human transmission through close contact; 77.4% of children had a history of household contact, and the remaining (22.6%) COVID-19 patients had an unknown exposure history and might have acquired community infection.

In this study, more than half of the paediatric COVID-19 patients were diagnosed with mild or moderate cases, and 1.61% of cases were severe, which was significantly less than the number of severe influenza cases. Additionally, the severity of COVID-19 in children was obviously different from that in adult patients. Among adult COVID-19 patients, 20 to 25% were admitted to the ICU, 5% had critical cases, and 3 to 4% died [13–15]. This might indicate that the pathogenicity of SARS-CoV-2 in children resembled that of two other severe-respiratory-disease-associated coronaviruses, severe acute respiratory syndrome coronavirus (SARS-CoV) and Middle East respiratory syndrome coronavirus (MERS-CoV), and children with SARS or MERS also suffered much less than adults with these illnesses [16, 17].

Fever and cough were the predominant symptoms at the onset of illness for both COVID-19 and influenza paediatric patients. Fever was identified in 34.68% of the COVID-19 cases, 70.21% of the influenza A cases and in 74.50% of the influenza B cases. Cough was present in 31.85% of COVID-19 cases, which was more than half of that in influenza cases. Although COVID-19 patients were less likely to exhibit symptoms and signs of respiratory infection, such as fever, cough, chills, sneezing, nasal congestion and rhinorrhoea, but more likely to present gastrointestinal symptoms, including vomiting, diarrhoea, dyspnoea and abdominal pain, it is not easy to differentiate COVID-19 from influenza in children on the basis of symptoms. Therefore, it might be necessary to closely monitor and examine child contacts.

The limitation of this study is that symptom-free children in the COVID-19 group were routinely screened out on the basis of close child contacts, but influenza patients were admitted to the hospital after the appearance of symptoms. This would upwardly bias the divergences of the COVID-19 group from the influenza groups. Another limitation is that COVID-19 patients were diagnosed on the basis of SARS-CoV-2 positivity in RT-PCR, but influenza patients were diagnosed on the basis of the presence of IgM antibody against influenza A virus or influenza B virus, which did not allow the comparison of COVID-19 and influenza cases at similar stages of disease duration. Partial information of the COVID-19 patients in this hospital centre was reported by other groups [7, 18, 19]. However, we independently described the characteristics of COVID-19 patients from different aspects. In addition, the oxygen saturation level was not detected in every patient. Only patients with moderate, severe and critical cases had oxygen saturation level data; therefore, we did not analyse these data. We could not obtain an accurate duration of oxygen support; therefore, we did not analyse these data or show this information in the tables.

## Conclusions

Similar to adult patients [20], there were many differences in clinical presentations between paediatric COVID-19 and influenza. Paediatric patients with COVID-19 had a lower severity of illness than children with influenza. The clinical presentations and outcomes were more favourable in paediatric COVID-19 patients than in paediatric influenza patients.

## Data Availability

The data were used under license for the current study, and so are not publicly available.

## Abbreviations

IQR: Interquartile Range
LOS: Length of Stay
SARS-CoV: Severe Acute Respiratory Syndrome Coronavirus
SARS-CoV-2: Severe Acute Respiratory Syndrome Coronavirus 2
MERS-CoV: Middle East Severe Acute Respiratory Syndrome Coronavirus
hsCRP: Hypersensitive C-reactive protein
ICU: Intensive Care Unit
RT-PCR: Real-time reverse transcription Polymerase Chain Reaction
MODS: Multiple Organ Dysfunction Syndrome
Flu: Influenza
IgM: Immunoglobulin M
